# General Anaesthesia by Intravenous Injection of Ether-Salt Solution

**Published:** 1913-04

**Authors:** 


General Anaesthesia by Intravenous Injection of Ether-Salt Solution.—L. W. Jenkins, Portland, Ore., *N. Y. Med.,* Dec., 1912. A saturated (7%) solution of ether in sterile physiologic salt solution is used, at body temperature. Allowing for evaporation, the solution actually contains about 5% of ether as injected. The stimulant stage begins when 25 c.c. have been injected, is not violent, and full narcosis is secured when 250-500 c.c. have been used. The average operation requires about 1-1½ liters for an hour, little more for two hours, the maximum employed being two liters, without untoward effect. Infection, embolism, overdistention of vessels and haemolysis are possible dangers but easily avoided. A rather complicated apparatus is illustrated by the author. The chief advantages are avoidance of disagreeable symptoms during the stimulant stage, mucous secretion and post-anaesthetic vomiting; and convenience and diminished risk of sepsis by eliminating the anaesthesia apparatus and administrator in head and neck operations. The anaesthetic is readily controled, small amounts are used and the method has given good practical results.

